# Parents' Awareness, Knowledge, and Perception of Amblyopia in Children: A Study in Jazan Region, Saudi Arabia

**DOI:** 10.7759/cureus.48956

**Published:** 2023-11-17

**Authors:** Ismail I Abuallut, Khalid M Alameer, Bandar M Abuageelah, Eman Hurissi, Masoud M Alqahtani, Ibrahim M Gosadi, Faisal M Tubaigy, Yousef M Alyami

**Affiliations:** 1 Department of Surgery, Ophthalmology Division, Jazan University‏‏, Jazan, SAU; 2 Department of Medicine, Jazan University, Jazan, SAU; 3 Department of Medicine and Surgery, Batterjee Medical College, Aseer, SAU; 4 Department of Medicine and Surgery, Jazan University, Jazan, SAU; 5 College of Medicine, University of Bisha, Bisha, SAU; 6 Department of Family and Community Medicine, Jazan University, Jazan, SAU; 7 Department of Ophthalmology, Faculty of Medicine, Jazan University‏, Jazan, SAU

**Keywords:** saudi arabia, jazan, awareness, knowledge, amblyopia

## Abstract

Background

Amblyopia is a vision disorder that results from a development problem in the brain rather than a neurological disease in the eye. The condition causes reduced visual acuity in one or both eyes due to the brain's inability to process inputs, rather than a structural abnormality. This study aims to assess parents’ awareness, knowledge, and perception of amblyopia in their children in the Jazan region, Saudi Arabia.

Methods

This is a cross-sectional study that used an electronic questionnaire consisting of five main sections to assess the level of awareness of amblyopia among parents in the Jazan region. The Statistical Package for Social Sciences (SPSS v.25, IBM Corp., Armonk, NY, USA) was utilized to input the data. The degree to which participants' level of awareness was related to variables such as age, gender, and educational level was assessed through the chi-square test (χ2), and any values that scored p-values under 0.05 were deemed statistically significant.

Results

The analysis included 572 participants, 395 mothers (69.0%) and 177 fathers (31.0%). The age groups of the participants were divided into 36-45 years (38.0%), 26-35 years (36.0%), and above 46 years (17.0%). Out of the total, 36 participants (6.0%) had a history of amblyopia, and 73 (13.0%) had a child who suffered from amblyopia. The findings showed that only 18 participants (3.1%) had a good awareness of amblyopia, while 242 (42.3%) had a fair level of awareness. Most participants, 312 (54.5%), were classified as having a poor awareness level of amblyopia. Parents with postgraduate degrees, those with prior awareness, and parents whose children had eye diseases demonstrated higher levels of good awareness. However, gender, age, and residency did not have significant associations with awareness levels.

Conclusion

While parents must be involved in managing amblyopia, our research found that more than half of the parents surveyed had a limited understanding of various aspects of the disease, which can cause permanent damage to their child's vision. Therefore, we recommend implementing health education programs to increase awareness and knowledge about amblyopia in Jazan.

## Introduction

Amblyopia, often known as a lazy eye, is derived from the Greek words ambles (dull) and ops (eye), describing a dullness of vision. It is a development problem in the brain, not an inherent neurological disease related to the eye. It describes a visual impairment in which the brain malfunctions in the processing of inputs, resulting in decreased best-corrected visual acuity in one or both eyes that cannot be linked to a structural abnormality [1-4]. It is commonly a monocular condition but can also be binocular without specific physical or pathological defects [5,6]. Deprivation, anisometropia, and strabismus are the most common causes of monocular amblyopia [7]. High uncorrected refractive errors mainly cause binocular amblyopia. A reduction in visual acuity primarily characterizes amblyopia. Still, it can also be accompanied by additional visual defects, including poor accommodation, binocular dysfunction, abnormal contour interactions, positional uncertainty, reduced contrast sensitivity, spatial distortions, abnormal eye movements, suppression, and fixation instability [8-10]. It is considered the most common cause of visual impairment in children [11-13]. Children between infancy and eight are most susceptible [14,15]. Treatment for amblyopia involves patching the healthy eye to encourage the use of the afflicted eye, and atropine has been shown in specific trials to relieve symptoms significantly [16-18]. However, the effectiveness of treatment declines beyond the age of eight if it is not started before the development of central vision [14,19]. The worldwide incidence of amblyopia has been estimated to be about 1.75% [20], whereas the total incidence of amblyopia among Saudi children is around 2.3% [21].

Amblyopia can impact the quality of life, leading to psychological complications such as depression and low self-esteem. In addition, it can cause poor school performance and foretell future employment challenges [22,23]. However, when the family and the community are involved in therapy, children with severe amblyopia can perform excellently in school [24]. When parents are unsure about the advantages of the treatment and are stressed or under relationship strain, they become involved in amblyopic treatment challenges [25]. Because amblyopia is predominantly a childhood disease, parents are generally the primary ones to recognize any changes or abnormalities in the appearance of their children's eyes, and they play a crucial part in ensuring adherence to their children's therapy plans and follow-up appointments with the ophthalmology clinic; therefore parents must be aware of the condition. For that, measuring parental awareness has significance because parents' lack of awareness of eye health establishes delays in getting necessary eye care at the proper time [26,27].

Amblyopia can have a negative influence on children's quality of life due to its effect on their learning process, ability to perform physical and social activities, and occupation choices. In addition, it can cause psychological complications. Parents' understanding of amblyopia and awareness of the importance of early diagnosis are critical in the prevention of visual impairment [28]. Therefore, this study assesses parents’ awareness, knowledge, and perception of amblyopia in their children in the Jazan region, Saudi Arabia, where this report is considered the first recent, detailed, special investigation on awareness of amblyopia among parents.

## Materials and methods

Study design

This cross-sectional study uses self-administered electronic questionnaires.

Sample

The sample size was determined using the Raosoft sample size calculator (Raosoft Inc., Seattle, WA, USA) based on the estimated population of the Jazan region, which is approximately 1,404,997 according to the General Authority for Statistics in the Kingdom of Saudi Arabia. With a 95% confidence interval, 5% margin of error, and 50% response distribution, the minimum sample size was established at 385.

Instruments

The validated survey, obtained from previous research [28,29], was divided into six sections. Section 1 covered demographics and personal information, section 2 evaluated amblyopia awareness, section 3 assessed awareness of symptoms, diagnosis, and trusted sources of information, section 4 measured knowledge of treatment options, section 5 evaluated attitudes towards amblyopia complications, and section 6 focused on parental awareness and support. Moreover, the survey was limited to individuals who resided in the Jazan region and had children, as confirmed by two screening questions: “Are you living in the Jazan region?” and “Do you have children?”. Only those meeting these criteria were permitted to participate in the survey.

Procedure

This study included participants who met the following criteria: they were 18 years old or older, lived in the Jazan region, and there were no gender restrictions, meaning males and females were both included. Participants of all nationalities (Saudis and non-Saudis) were included, provided they consented to participate in the survey. However, individuals under 18 years old, those suffering from chronic debilitating diseases that made it impossible to partake in the survey, those who declined to participate or did not finish the questionnaires, and those who were illiterate or unable to use technology were excluded from the study.

Approval for the study was obtained through the research ethics committee at Jazan University (approval number: REC-44/09/594) on 30 March 2023. After outlining the objectives of the study, participants electronically consented. The survey was uploaded online and was accessible without any login restrictions; it was promoted using all accessible social media (e.g., WhatsApp, Telegram, and Twitter).

Statistical analysis

The study aimed to measure awareness of amblyopia, scoring responses using binary coding criteria. Correct responses received a one, while incorrect ones received a zero. Multiple-choice questions were scored based on selecting at least half of the correct options. Participant awareness was classified as poor, fair, or good based on their total score of 15. Using this score, we divided the participants into three groups based on their level of awareness: good, fair, and poor. If a participant answered 0-7 questions correctly, they were considered to have a poor level of awareness. If they answered 8-11 questions correctly, they were classified as having a fair level of awareness, while those who answered 12-15 questions correctly were considered to have a good level of awareness.

For the analysis of the question regarding the level of perception and attitude about amblyopia complications and impact and the role of parents (fifth and sixth sections), we used the Likert scale [30] in which participants specify their level of agreement and importance for a series of statements in fifth and sixth sections respectively. Data analysis included descriptive statistics and a chi-square test (χ2) to examine the association between qualitative variables, specifically the three-level awareness score. A P-value less than 0.05 was considered statistically significant, and SPSS v.25 (IBM Corp., Armonk, NY, USA) was used for all analyses [31].

## Results

Sociodemographic characteristics

A total of 572 responses were analyzed in the current study. The majority of parents were females (69%), married (92%), and had at least one child (52%). The most common educational level was a bachelor's degree (56%). The majority of parents had never been diagnosed with amblyopia (94%) or had any eye diseases (68%). However, 29% of parents reported that one of their children had eye disease, and 13% reported that one of their children had amblyopia. More details about the sociodemographic characteristics are listed in Table 1.

**Table 1:** Socio-demographic variables.

Variable		Frequency	Percentage
Gender	Male	177	31%
	Female	395	69%
Age groups	25 and younger	54	9%
	26-35	204	36%
	36-45	216	38%
	46 and older	98	17%
Residency	Urban	268	47%
	Rural	304	53%
Marital status	Married	525	92%
	Divorced	34	6%
	Widowed	13	2%
Occupation	Student	40	7%
	Employee	319	56%
	Unemployed	33	6%
	Retired	27	5%
	Housewife	153	27%
Educational level	Less than university	133	23%
	Diploma	79	14%
	Bachelor	321	56%
	Higher education or Postgraduate	39	7%
Number of children	No children	46	9%
	1-3	295	52%
	4-6	181	32%
	7 and more	50	9%
Do any of your children have eye diseases?	Yes	164	29%
	No	408	71%
Have you been diagnosed with amblyopia before?	Yes	36	6%
	No	536	94%
Do you or your partner have any eye diseases?	Yes	184	32%
	No	388	68%
Do any of your children have amblyopia?	Yes	73	13%
	No	499	87%
Have you ever heard of amblyopia?	Yes	292	51%
	No	280	49%

Participants’ awareness towards knowledge items of amblyopia

The highest proportions of correct answers were provided for the items indicating the importance of examining the child's visual acuity before school entrance to ensure the normal development of vision (86%), most cases of amblyopia are discovered accidentally (82.7%), and the practice of parents concerning taking children for a routine vision screening (71.3%). Conversely, the lowest proportions of correct answers were for the items regarding the causes and definition of amblyopia (0.7% and 4.7%, respectively) and the recommended number of vision screenings for a child aged 6 to 12 years (7.5%). Detailed descriptive analyses regarding the correct answers are demonstrated in Table 2. Assessment of knowledge categories revealed that more than half of respondents had a poor level of knowledge (54.4%), whereas only 3.1% had a good knowledge level (Table 3).

**Table 2:** Participants’ responses towards awareness of amblyopia.

Awareness	Variable	Correct Answers	
		Percent	Frequency
	What is the definition of amblyopia?	4.7%	27
	What are the causes of amblyopia?	0.7%	4
	Which age group can amblyopia affect?	23.6%	135
	Is it unnecessary to have an eye examination for a child whose eyes appear healthy?	33.9%	194
	Amblyopia can be diagnosed by a General Paediatric or family doctor?	30.6%	175
	Because the majority of cases are discovered by chance, is it necessary for an ophthalmologist to screen and examine the child's eye?	82.7%	473
	Is closing the eyes for a short time or pressing them while watching TV a sign indicating the possibility of amblyopia?	49.1%	281
	Is it difficult for parents to notice this problem because the child is unable to recognize that his or her vision is weak?	52.6%	301
	Is it essential to examine the child’s visual acuity before school entrance to ensure normal vision development?	86%	492
	What is the recommended number of vision checks for a 6- to 12-year-old child?	7.5%	43
	Do you take your child for a routine vision screening?	71.3%	408
	Is there a treatment for amblyopia?	51.7%	296
	Do you think the treatment must be at an early age?	68%	389
	What is the best age range to treat amblyopia?	41.6%	238
	Do you think that if amblyopia is not treated at a young age, it will worsen?	77.1%	441

**Table 3:** Level of awareness of amblyopia among parents in Jazan, Saudi Arabia.

Level of awareness	Frequency	Percent
Poor	312	54.5%
Fair	242	42.3%
Good	18	3.1%

The relationship between the sociodemographic characteristics of parents and their awareness levels regarding amblyopia

Results showed a significant difference in awareness levels based on parents’ educational levels (p < 0.001), where parents with postgraduate degrees exhibited the highest proportion of good awareness (10.26%), followed by those with less than university education (2.26%), bachelor's degree holders (2.8%) and diploma holders (2.53%). In terms of prior awareness, participants who had heard of amblyopia before demonstrated a significantly higher proportion of good awareness (4.11%) compared to those who had not (2.14%, p < 0.001). Additionally, parents whose children had eye diseases showed a notably higher proportion of good awareness (4.27%) than those without affected children (2.7%, p < 0.001). However, no significant associations were found between awareness levels and participants' gender, age groups, or residency (Table 4).

**Table 4:** The relationship between “awareness levels of amblyopia among parents” and “participants variables”. *statistically significant (p-Value < 0.05)

Level of awareness					
Variable		Poor, n (%)	Fair, n (%)	Good, n (%)	p-Value
Gender	Male	101 (57.6%)	70 (39.55%)	6 (3.39%)	0.668
	Female	211 (53.42%)	172 (43.54%)	12 (3.04%)	
Age groups	25 and younger	35 (63.8%)	17 (31.48%)	2 (3.7%)	0.168
	26-35	118 (57.84%)	77 (37.75%)	9 (4.41%)	
	36-45	112 (51.85%)	100 (46.3%)	4 (1.85%)	
	46 and older	47 (47.96%)	48 (48.98%)	3 (3.06%)	
Residency	Urban	140 (52.24%)	117 (43.66%)	11 (4.1%)	0.337
	Rural	172 (56.58%)	125 (41.12%)	7 (2.3%)	
Educational level	Less than university	91 (68.42%)	39 (29.32%)	3 (2.26%)	< 0.001*
	Diploma	43 (54.43%)	34 (43.04%)	2 (2.53%)	
	Bachelor	166 (51.71%)	146 (45.48%)	9 (2.8%)	
	Higher education or Postgraduate	12 (30.77%)	23 (58.97%)	4 (10.26%)	
Have you ever heard of amblyopia?	Yes	126 (43.15%)	154 (52.74%)	12 (4.11%)	< 0.001*
	No	186 (66.43%)	88 (31.43%)	6 (2.14%)	
Do any of your children have eye diseases?	Yes	61 (37.2%)	96 (58.54%)	7 (4.27%)	< 0.001*
	No	251 (61.52%)	146 (35.78%)	11 (2.7%)	

Perception about amblyopia complications

Table 5 shows the proportions of parents who agreed or disagreed with statements about the complications and impact of amblyopia. The complication with the highest proportion of awareness was decreased visual acuity, with 95.7% of parents agreeing or strongly agreeing that this was a possible complication. Other complications that were commonly perceived to be associated with amblyopia (agreed or strongly agreed) included disability (89%), school failure (87.9%) and an impaired quality of life (83.2%). On the other hand, less than half of the participants disagreed or strongly disagreed that amblyopia may be complicated by blindness (48.1%) and an economic burden on the family (44.4%).

**Table 5:** Perception about amblyopia complications.

Perception about Amblyopia complications and impact	Strongly disagree		Disagree		Agree		Strongly agree	
	Frequency	%	Frequency	%	Frequency	%	Frequency	%
Decreased visual acuity	4	0.7%	21	3.7%	232	40.6%	315	55.1%
Double vision	30	5.2%	113	19.8%	225	39.3%	204	35.7%
Blindness	59	10.3%	216	37.8%	167	29.2%	130	22.7%
Disability	20	3.5%	43	7.5%	284	49.7%	225	39.3%
Stigmatization	38	6.6%	81	14.2%	236	41.3%	217	37.9%
Impaired quality of life	29	5.1%	67	11.7%	261	45.6%	215	37.6%
School failure	23	4.0%	46	8.0%	265	46.3%	238	41.6%
Adverse impact on the family	59	10.3%	134	23.4%	215	37.6%	164	28.7%
Economic burden (family)	87	15.2%	167	29.2%	184	32.2%	134	23.4%

The perceived importance of parents’ roles in various dimensions of amblyopia

As demonstrated in Table 6, the highest proportion of participants found the parents’ role is important or very important in follow-up (96.3%), treatment compliance (96%), early detection (94.3%), and social support (94.1%). Contrastingly, the role of parents in amblyopia prevention was important or very important as expressed by 84.1% of parents (Table 6).

**Table 6:** Parents' roles in amblyopia were perceived in various dimensions. A: important or very important (percentage).

Statement	Not important at all		Not important		Somewhat important		Important		Very important		A
Parents play a role in	Frequency	%	Frequency	%	Frequency	%	Frequency	%	Frequency	%	%
Amblyopia prevention	6	1.0%	16	2.8%	69	12.1%	162	28.3%	319	55.8%	84.1%
Early detection	2	0.3%	5	0.9%	26	4.5%	133	23.3%	406	71.0%	94.3%
Diagnosis	2	0.3%	10	1.7%	33	5.8%	141	24.7%	386	67.5%	92.2%
Treatment efficacy	4	0.7%	4	0.7%	38	6.6%	143	25.0%	383	67.0%	92.0%
Treatment compliance	2	0.3%	3	0.5%	18	3.1%	121	21.2%	428	74.8%	96.0%
Follow-up	4	0.7%	2	0.3%	15	2.6%	123	21.5%	428	74.8%	96.3%
Social support	3	0.5%	5	0.9%	26	4.5%	156	27.3%	382	66.8%	94.1%
Psychological support	2	0.3%	3	0.5%	23	4.0%	127	22.2%	417	72.9%	95.1%

Sources of information regarding amblyopia

**Figure 1:**
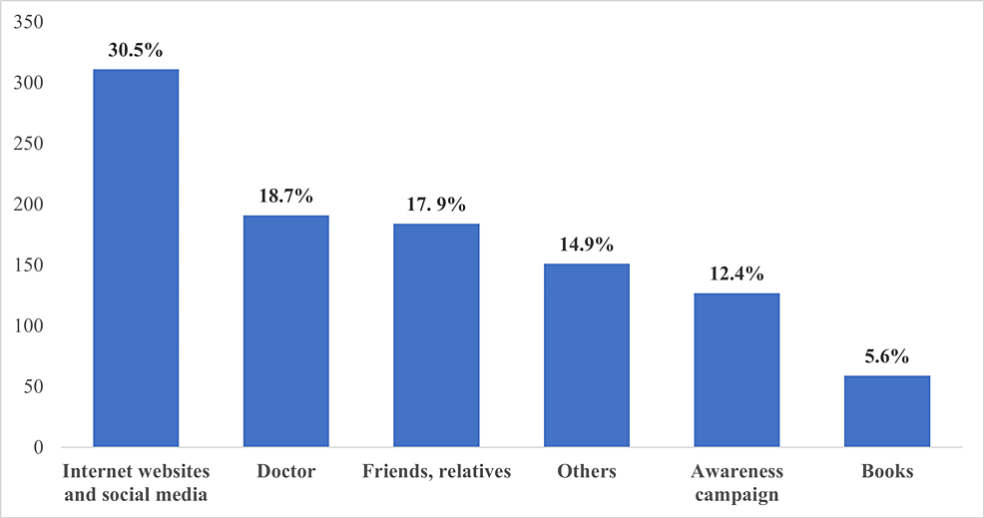
The most common sources of information regarding amblyopia.

Of note, the most common source of information among parents included the internet and social media platforms (30.4%), doctors (18.7%) and friends or relatives (18.0%), whereas books and awareness campaigns represented the least common sources of information (5.8% and 12.4%, respectively, Figure 1).

## Discussion

Early management of amblyopia is vital as the vision develops in the first few years of life. Inadequate knowledge level regarding eye screening and warning eye symptoms and signs lead to delays in the diagnosis and management. Parents' awareness is the key to early diagnosis and management of amblyopia in children.

In this study, the knowledge and awareness levels among parents were assessed and it showed that more than half of the respondents had a poor level of knowledge (54.5%). Many studies conducted in different regions of Saudi Arabia over several years have shown similar results to our study. A study conducted by Aljohani et al. in 2016, in Jeddah, aimed to assess common eye diseases knowledge and awareness among the Saudi population. The results of the study showed that only 10% of the participants had heard about amblyopia [32]. Another study conducted by Basheikh et al. in 2016, at Red Sea Mall, Jeddah, aimed to assess the knowledge and awareness levels among parents and companions in Jeddah. The results showed a low-level knowledge (25.9%) [33]. In 2019, a study conducted by Alsaqr and Masmali aimed to investigate parents’ awareness of amblyopia in different regions of Saudi Arabia. The study results showed that 70% of the participants had no previous knowledge of amblyopia [34]. Our study showed a higher level of amblyopia awareness and knowledge (45.4%) compared to the findings of Aljohani et al. [32], Basheikh et al. [33] and Alsaqr and Masmali [34], and these results are somehow expected due to the years gap and the efforts of the awareness campaign and health education over the years. However, a better level of awareness and knowledge has been noticed in a study conducted in 2018 by Alzahrani et al. that aimed to evaluate strabismus knowledge and awareness; the target population was the parents and children companions who attended the pediatrics and ophthalmology clinics. The results showed that awareness of amblyopia was 50% [35]. Since Alzahrani's study was conducted in the hospital setting, the awareness level is expected to be higher than Aljohani et al. [32] and Basheikh et al. [33] studies which were conducted mainly in the mall, and Alsaqr and Masmali [34] and our study in which the questionnaire was distributed online with higher chance of selection bias and responder bias. However, our results were higher than Mocanu and Horhat study in which amblyopia was identified by 2.8% of the participants [36]. Regarding the knowledge about amblyopia causes, our study showed that the level of knowledge was 0.7%, similar to the results reported by Basheikh et al. study (0.0%) [33]. Higher identification of amblyopia causes was found in Alhaddab et al. study where 28.6% and 10.0% of participants identified refractive error and cataract as the most common causes of amblyopia [29]. Moreover, our study showed statistically significant results in the awareness levels of the parents who have postgraduate degrees, who heard of amblyopia before, and whose children had eye diseases when compared to their counterparts. This result is consistent with Alshaheen and Al Owaifeer's study which found a significant increase in the awareness and perception level among parents who had a Master's or Ph.D. degree and those who have a family history of amblyopia [37]. In our study, the complications with the highest proportion of awareness were decreased visual acuity, disability, school failure and impaired quality of life, respectively. These results are similar to Aljohani et al. [32] and Alhaddab et al. results [29]. Also, in our study, the most common sources of information regarding amblyopia were the internet and social media platforms (30.5%) and the doctors (18.7%) which is consistent with the findings of Alsaqr and Masmali [34] and Basheikh et al. [33]. In addition, our study showed that the parents showed very high awareness regarding the importance of their roles in the follow-up, treatment compliance, early detection, social support and prevention. These results are consistent with Alhaddab et al. [29] and Alzahrani et al. studies [35].

The findings may have limitations because the data was collected through self-reporting, which could result in an accurate classification bias if some participants need to understand the questions. The study could also have introduced another bias when participants could select multiple answers, resulting in a false score if all the options were chosen. Moreover, there is a possibility of selection bias in the sample as it comprises a more significant proportion of females than males. However, this can be attributed to the fact that there is a higher population of females than males in the Jazan region.

## Conclusions

Amblyopia management depends primarily on the parents' awareness and knowledge of eye care and the warning signs and symptoms that can affect their children's vision. Additionally, parental care is vital throughout the different management stages. Our study showed that more than half of the parents needed a better level of knowledge regarding different aspects of the disease, which can affect the child's vision permanently.

To improve awareness of amblyopia among the Jazan population, the governmental health entities, e.g., the Ministry of Health, should organize community outreach programs, collaborate with schools, and offer free eye check-ups to encourage early detection of amblyopia, which can help in the prevention of long-term vision problems. Spreading knowledge through social media, partnering with community leaders, and educating people on the condition's symptoms and treatment options are also crucial.

## Appendices

Section 1 Socio-demographic & awareness of amblyopia:

It contains demographic information such as age, gender, marital status, career and educational level, place of residence, and awareness about amblyopia and its diagnosis.

Participant’s characteristics

Gender:

·        Male

·        Female

Residency:

·        Urban

·        Rural

Age:

      .................................

Marital status:

·        Married

·        Divorced

·        Widowed

Occupation:

·        Student

·        Employee

·        Unemployed

·        Retired

·        Housewife

Educational level:

·        Less than university

·        Diploma

·        Bachelor

·        Higher education or Postgraduate.

Number of children:

.................................

Do you or your partner have any eye diseases?

·        Yes

·        No

Do any of your children have eye diseases?

·        Yes

·        No

Have you been diagnosed with amblyopia before?

·        Yes

·        No

Do any of your children have amblyopia?

·        Yes

·        No

Awareness about amblyopia and its diagnosis

Have you ever heard of amblyopia?

·        Yes

·        No

What is the definition of amblyopia? (You may select more than one choice.)

·        An imbalance in the movement of one eye

·        It is a weakness in the vision ability in one eye or both without underlying pathology

·        It occurs as a result of the brain ignoring the blurred image of the amblyopic eye

·        The eye is anatomically normal, but not functioning properly

·        Decreased night vision

·        Involuntary eye movement

·        Over time, the brain relies only on the healthy eye

What are the causes of amblyopia? (You may select more than one choice.)

·        Hereditary

·        Refractive defects (near or farsightedness, astigmatism)

·        Sunlight exposure

·        Glaucoma

·        Malnutrition

·        Strabismus

·        Cataract

·        Cerebral palsy

·        Electronic devices

·        Fever during childhood

·        Premature infant

·        Upper eyelid drooping

·        Unknown reasons

Which age group can amblyopia affect?

·        Children

·        Adult

·        All of the above

Awareness about amblyopia diagnosis

·        Is it unnecessary to have an eye examination for a child whose eyes appear healthy? Agree

·        Not Agree

·        I don't know

Amblyopia can be diagnosed by General Pediatric or family doctor?

·        Yes

·        No

·        I don’t know

Is closing the eyes for a short time or pressing them while watching TV a sign indicating the possibility of amblyopia? Agree

·        Not Agree

·        I don't know

Is it difficult for parents to notice this problem because the child cannot know that his/her vision is weak?

·        Agree

·        Not Agree

·        I don't know

Is it essential to examine the child’s visual acuity before school entrance to ensure the normal development of vision?

·        Agree

·        Not Agree

·        I don't know

Because the majority of cases are discovered by chance, is it necessary for an ophthalmologist to screen and examine the child's eye? Agree

·        Not Agree

·        I don’t know

What is the recommended number of vision screenings for a child aged (6-12) years?

·        Annually

·        Every two years

·        Only when there are symptoms

·        Once only

·        I don't know

Do you take your child for a routine vision screening?

·        Yes, annually

·        No, only if he complains about something

·        No, just before he entered the school

What are your sources of information on Amblyopia? (You may select more than one choice.)

·        Friends, relatives

·        Internet websites and social media

·        Doctor

·        Awareness campaign

·        Books

·        Others

Section 2 Perception and attitude about amblyopia complications and treatment outcomes:

Perception about treatment outcomes

Is there a treatment for amblyopia?         

·        Yes

·        No

·        I don’t know

Do you think the treatment must be at an early age?

·        Yes

·        No

·        I don’t know

What is the best age range to treat amblyopia?

·        After the age of 10 years

·        Before the age of one year

·        Between the period of 3 to 9 years

·        There is no specific age period

Do you think amblyopia becomes worse if left untreated at an early age?

·        Yes

·        No

·        I don’t know

One of the complications of amblyopia that it may cause

Decreased visual acuity

·        Strongly disagree

·        Disagree

·        Agree

·        Strongly agree

Double vision

·        Strongly disagree

·        Disagree

·        Agree

·        Strongly agree

Blindness

·        Strongly disagree

·        Disagree

·        Agree

·        Strongly agree

Disability

·        Strongly disagree

·        Disagree

·        Agree

·        Strongly agree

Stigmatization

·        Strongly disagree

·        Disagree

·        Agree

·        Strongly agree

Impaired quality of life

·        Strongly disagree

·        Disagree

·        Agree

·        Strongly agree

School failure

·        Strongly disagree

·        Disagree

·        Agree

·        Strongly agree

Adverse impact on the family

·        Strongly disagree

·        Disagree

·        Agree

·        Strongly agree

Economic burden (family)

·        Strongly disagree

·        Disagree

·        Agree

·        Strongly agree

Section 3 Perceived parents’ role in amblyopia in different dimensions:

Parents play a role in

Amblyopia prevention

·        Not important at all

·        Not important

·        Somewhat important

·        important

·        Very important

Early detection

·        Not important at all

·        Not important

·        Somewhat important

·        important

·        Very important

Diagnosis

·        Not important at all

·        Not important

·        Somewhat important

·        important

·        Very important

Treatment efficacy

·        Not important at all

·        Not important

·        Somewhat important

·        important

·        Very important

Treatment compliance

·        Not important at all

·        Not important

·        Somewhat important

·        important

·        Very important

Follow-up

·        Not important at all

·        Not important

·        Somewhat important

·        important

·        Very important

Social support

·        Not important at all

·        Not important

·        Somewhat important

·        important

·        Very important

Psychological support

·        Not important at all

·        Not important

·        Somewhat important

·        important

·        Very important
 
